# Impact of polyacrylamide supplementation on intake, nutrient digestion and growth of lambs

**DOI:** 10.1371/journal.pone.0284509

**Published:** 2023-04-20

**Authors:** Zhixiang Zhai, Qiujiang Luo, Yong Chen, Ron Pan, Changjiang Zang

**Affiliations:** Laboratory of Animal Nutrition, Xinjiang Agricultural University, Urumqi, China; University of Agriculture Faisalabad, PAKISTAN

## Abstract

This study was conducted to evaluate the effect of polyacrylamide (PAM) supplementation on the intake, digestion, weight gain, metabolism and growth of lambs. A total of ten 30 days old male small-tailed Han lambs with a body weight of 7.7±0.5 kg were divided into two equal groups (n = 5 each) and fed a basal diet or diet supplemented with 2.0 g of PAM per kg diet. The duration of the experiment was 210 days and experimental diets were fed *ad libitum* throughout the experimental period. Voluntary feed intake (VFI) was measured on daily basis, while body weight was measured on every ten days of the experiment.Two digestive and metabolic trials were conducted at the lamb’s age of 95 to 103 days (Trial 1) and at the age of 210 to 218 days (Trial 2). At the end of experiment, all lambs were slaughtered to determine carcass characteristics. Results of the current study showed that supplementation of PAM in the diet of lambs increased the VFI and daily body gain by 14.4% (*P <* 0.05) and 15.2% (*P <* 0.01), respectively. In Trial 1, PAM supplementation in the diet increased the digestibility of dry matter (DM), organic matter (OM), crude protein (CP), cellulose, energy, and nitrogen retention by 7.9%, 5.4%, 6.4%, 9.6%, 4.3% and 30.3% (*P <* 0.01), respectively, and in Trial 2, PAM supplementation in the diet increased the digestibility of DM, OM, CP, cellulose, energy, and nitrogen retention by 9.3%, 7.9%, 7.7%, 11.6%, 6.9% and 38.5% (*P <* 0.01), respectively. Results of carcass parameter explored that supplementation of PAM in the diet increased the carcass, net meat and lean meat weights by 24.5%, 25.5%, and 30.6% (*P <* 0.01), respectively, however, PAM supplementation in the diet did not influence the contents of DM, OM, or CP in fresh liver, leg muscle, and rumen tissue; in addition, the CP contents in the *Longissimus dorsi* muscle was decreased by the supplementation of PAM in the diet. In summary, supplementation of 2.0 g of PAM per kg diet increased the VFI, nutrient digestibility, nitrogen retention, and carcass yield of lambs.

## Introduction

Ruminants have a valuable role in sustainable agricultural systems and provision of food to human beings. They play a pivotal role in converting vast renewable resources from rangeland, pasture, and crop residues and/or other by-products into food edible with the help of rumen. The rumen of the ruminants harbors complex microbiota, including bacteria, protozoa, archaea, and fungi, which together play pivotal roles in transforming plant material into exploitable nutrients [1, 2]. It has been reported previously that manipulation of rumen microbes and their metabolism can affect the productivity of animals [3–5].

Improving the production performance of animal by regulating rumen fermentation has always been a topic of interest in ruminant nutrition research. Previous studies have reported that surfactants have ability to manipulate the rumen microbial community, ruminal fermentation pattern and hence the productivity of the animals [6–9]. For example, the addition of the well-known nonionic surfactant, alkyl polyglycoside (APG) in the diet has been reported to enhance the utilization of fatty acids by rumen bacteria and change in functions of bacteria of goats [6]. Similarly, another study has reported that inclusion of APG into the diets of lactating cows linearly increase the non fat solid and total solid contents in milk [7]. A study on sheep also demonstrated that addition of the nonionic surfactants Tween 60 and Tween 80 in the diet of sheep influence the rumen metabolites and results in the reduction of ammonia nitrogen, and increase in the concentration of volatile fatty acids without affecting voluntary feed intake (VFI) and digestion [8].

Surfactant not only influence the rumen metabolites but have impact on rumen microflora. For example, a recent study has demonstrated that the biosurfactant manosylerythritol lipid decrease the numbers of most of ruminal gram-positive bacteria, reduce methane production, and increase the ratio of propionate to acetate [10]. Our previous studies have extensively explored the anionic surfactant docusate (DOC or aerosol OT), an anionic surfactant, led to a in the diet decreased protozoan count, increased total amount of bacteria in rumen [11], increased activities of fiber-degrading enzymes in the rumen [12], increased nutrient absorption from the small intestine [9] and nitrogen retention in sheep [13, 14], and improved the slaughter performance of lambs [7]. Our recent study had also explored that the DOC supplementation not only affects feed intake and digestion of cattle but also improve nitrogen retention of cattle [15]. Based on the findings of previous studies and results of our in vivo trials it could be assumed that DOC have ability to alter ruminal microflora, ruminal metabolites, ruminal digestion and metabolism and hence the productivity of ruminants.

Polyacrylamide (PAM) is an anionic surfactant similar to DOC that is often used as a chemical assistant in oil fields [16], as a clarificant in water factories [17], or as a clarificant in fruit juice [18]. Our previous study have successfully supplemented PAM at 2.0 g/kg diet in sheep and explored that supplementation of PAM at 2.0 g/kg diet in sheep impact on the ruminal microbial flora and metabolism of sheep, and it increase VFI, enhance fiber-degrading enzymes activity, total bacterial abundance and decrease the protozoan count in the rumen of sheep [19].

However, to our knowledge, none of the study have expored the impact of polyacrylamide supplementation on digestion, metabolism, growth and carcass characteristics of lambs. Therefore, in the present study, the effects of PAM supplementation at 2.0 g/kg diet on VFI, digestion and metabolism of nutrients and slaughter performance of growing lambs were examined to verify the possible positive effect of PAM on sheep.

## Materials and methods

### Animals and experimental design

The experimental protocol in this study were approved by the Animal Care Committee, Xinjiang Agricultural University (Urumqi, China), and the experimental procedures were in accordance with the University’s guidelines for animal research. A total of ten 30 days old weaned, small-tailed Han male lambs, with a body weight of 7.7±0.5 kg, were selected for the current experiment. Lambs were equally divided into two groups (n = 5) in such a way that each group fed one of the basal diet or a basal diet supplemented with 2.0 g of PAM per kg diet. All animals were kept in individual pens in an animal house with free access to fresh drinking water.

### The diets and management of animals

According to nutrient requirement of meat-type sheep and goat [20], the ingredients and nutritional compositions of the basic diets are listed in Table 1. The starter, grower, and finisher diets were offered to the lambs aged 30 to 80, 81 to 120, and 121 to 240 days, respectively. The percentages of ground cornstalk in the starter, grower, and finisher diets were 4.5%, 14.2% and 28.4%, respectively, and the contents of crude protein (CP) were 19.8%, 14.7% and 11.9%, respectively. The PAM dose was supplemented at 2.0 g/kg diet, mixed with the concentrate first, and then fed to the animals in the experimental group.

**Table 1:** The ingredients and nutritional compositions of the basal diets for lambs (%, dry matter basis).

Diets	Starter	Grower	Finisher
Lamb age (days)	30~80	81~120	121~240
Ingredients			
Ground cornstalk	3.12	14.41	28.93
Ground corn	62.29	59.13	55.70
Cottonseed meal	24.97	18.32	9.77
Cooked soybean flour	6.85	5.42	2.89
Limestone	0.268	0.216	0.203
Calcium hydrophosphate	0.002	0.004	0.007
Nutrient premix^1^	2.50	2.50	2.50
In total	100.00	100.00	100.00
Analyzed nutrient compositions^2^			
Organic matter	93.70	92.20	91.80
Crud protein	19.70	15.76	13.78
Cellulose	4.20	14.60	25.20
Hemi-cellulose	9.11	9.50	19.50
Calcium	1.03	0.96	1.01
Phosphorus	0.59	0.59	0.62
Gross energy (MJ/kg)	18.70	17.50	16.30

^1s^ The premix provides nutrients per kg of the basal diet: VA, 1350 IU; VD, 270 IU; VE, 45 IU; iron, 16 mg; copper, 8 mg; zinc, 5 mg; manganese, 10 m;, iodine, 8.5 mg; cobalt, 0.10 mg; and selenium, 0.20 mg.

^2^ The contents of nutrients were analyzed.

The lambs were weaned at the age of 30 days, raised in individual pens and fed *ad libitum*. Fresh drinking water was available all times. The animals were fed twice per day at 9:00 a.m. and 18:00 p.m., and the amount of diet offered was adjusted every day according to refusal on the previous day, ensuring that 3% to 5% of the feed was left to maintain a constant ratio of the concentrate to roughage.

The purchased PAM (food grade) was a white powder with a molecular weight range of 3 to 22 million Da, an ion density range of 10% to 50%, and solid content ≥ 90%.

### Collection, pretreatment and storage of samples

The amounts of feed offered and refusal before the morning feeding were collected and recorded daily for 210 days to calculate the VFI at 10-day intervals. The live body weight before the morning feeding was recorded every 10 days to calculate the ADG.

The digestive and metabolic trials were conducted twice at the ages of 95 to 103 days and 210 to 218 days. During each trial, the lambs were placed in metabolic cages for 11 days, including the first three days for adaptation and the remaining eight days for sample collection. During the period of sample collection, the feces and urine of each lamb were collected at 9:00 a.m. and the weights were recorded. A sample of 10% of feces or urine was taken. Fecal samples for each lamb were dried at 65°C and pooled in each trial. The urine sample was mixed with 2 mL of hydrochloride acid (1:1) and stored at -20°C. The amounts of diet offered and the refusal were sampled daily to analyze and calculate the nutrient intake, and the samples were stored in a refrigerator. At the end of each trial, all samples for an individual were pooled together and subsampled. The samples were dried at 65°C and ground to pass through a 60 mesh sieve for laboratory analyses.

At the end of the experiment, the lambs were fasted overnight, weighed, and then sacrificed. The carcass, carcass lean meat, carcass fat, carcass bones, leg muscle, *Longissimus dorsi* muscle, heart, liver, kidneys, and emptied rumen were all weighed. Fresh liver, kidney, emptied rumen, leg muscle, and *Longissimus dorsi* muscle tissues were sampled (approximately 100 g each) and stored at -20°C for further analysis. The rumen was rinsed with saline before being weighed, and a sample at 20 cm to 30 cm from the entrance of the rumen was obtained.

### Assays of samples and calculations

The samples of feed, refusal, feces, urine, liver and other tissues were analyzed using AOAC methods [21]: dry matter (DM, Code 930.15), organic matter (OM, Code 942.05), crude protein (CP, Code 990.03), calcium (Ca, Code 920.39), and phosphorus (P, Code 946.06). Gross energy was determined using an automatic adiabatic oxygen bomb calorimeter. The contents of neutral detergent fiber (NDF), acid detergent fiber (ADF), and acid detergent lignin (ADL) in feed and feces were determined using the procedures of van Soest *et al*. [22]. Hemicellulose and cellulose contents were calculated as NDF-ADF and ADF-ADL, respectively. Retention rates of nitrogen, calcium and phosphorus were calculated as follows:

$$\mathrm{Retention}\;\mathrm{rate}\;(\%)=\frac{\mathrm{intake}\;(g)‐\mathrm{fecal}\;\mathrm{excretion}\;(g)‐\mathrm{urinary}\;\mathrm{excretion}\;(g)}{\mathrm{intake}\;(g)}$$ (1)

### Measurement of DNA and RNA contents in tissues

The contents of DNA and RNA in the tissue samples were determined using the diphenylamine method [23] and orcinol [24], respectively, in which a correction of DNA interference in the RNA assay was induced by the orcinol method, namely, optical density (OD) of RNA = assayed OD of RNA—OD of RNA induced by DNA. Briefly, approximately 1 g of tissue sample was cut into tiny pieces by scissors, extracted with 2×3 mL of acetone to remove most of the fat, mixed with 10 mL of 12% trichloroacetic acid and digested in a boiling water bath for 15 minutes with interval disturbances. The supernatant was collected after centrifugation, and then two aliquots of diphenylamine reagent were mixed with one aliquot of supernatant and placed in a boiling water bath for a minute. After the solution was cooled in ice water, the OD_595_ value of the sample was determined. Additionally, the supernatant was mixed with two aliquots of orcinol reagent and placed in a boiling water bath for 20 minutes. After the solution was cooled in ice water, the OD_680_ of the sample was determined. At the same time, the OD_680_ of the DNA standard was assayed with the orcinol reaction when the DNA content (OD_595_) was assayed to account for the disturbance of DNA for calculating RNA. Calf thymus DNA sodium salt and yeast RNA were used to generate standard curves for DNA and RNA, respectively.

### Statistical analysis

The data are expressed as the mean value and standard error of the mean (SEM). The data were analyzed in a *t test* for paired samples by SPSS 17.0 software. Statistical significance was declared with a *P* value < 0.05 or < 0.01.

## Results

### Impact of supplementation of polyacrylamide on voluntary feed intake and daily body gain of lambs

As shown in Table 2, PAM supplementation increased the VFI of lambs aged 30–80, 81–120, and 121–240 days by 4.9% (*P* > 0.05), 14.4% (*P* < 0.01) and 16.0% (*P* < 0.01), respectively. The daily body weight gain (DBG) was consistently increased by PAM supplementation from the age of 30 days until 240 days and by 8.8%, 6.6% and 22.5% (all *P* < 0.01) at three stages of age (days). The feed conversion ratio (FCR) was no significant difference at the starter phase (*P* > 0.05), but it increased significantly at the grower phase (*P* < 0.01) and decreased significantly at the finisher phase (*P* < 0.05) by PAM supplementation. Over the whole period of 210 days, PAM supplementation increased VFI and DBG by 14.4% and 15.2% (both *P* < 0.01), respectively, while the FCR were 5.24 and 5.21 (g/g) for the control and PAM treatments (*P* > 0.05), respectively.

**Table 2:** The effect of polyacrylamide supplementation on the voluntary feed intake, daily body gain and feed conversion ratio of lambs.

Treatments	Control	^1^PAM	SEM	*P value*
Starter				
Initial ^2^BW at age 30 days (kg)	7.66	7.64	0.22	0.704
BW at age 80 days (kg)	15.36	16.02	0.71	0.003
^3^VFI (g/lamb/day)	477.6	501.0	65.40	0.162
^4^DBG (g/lamb/day)	154.0	167.6	10.56	0.002
^5^FCR (g/g)	3.06	2.96	0.23	0.412
Grower				
BW at age 120 days (kg)	25.70	27.04	0.98	<0.001
VFI (g/lamb/day)	764.4	874.2	71.56	<0.001
DBG (g/lamb/day)	258.5	275.5	7.35	0.005
FCR (g/g)	2.95	3.17	0.22	<0.001
Finisher				
BW at age 240 days (kg)	44.68	50.30	0.73	<0.001
VFI (g/lamb/day)	1164.8	1351.2	41.23	<0.001
DBG (g/lamb/day)	158.2	193.8	3.93	<0.001
FCR (g/g)	7.40	7.00	0.39	0.044
Overall average from age 30 to 240 days				
VFI (g/lamb/day)	924.9	1057.9	52.50	<0.001
DBG (g/lamb/day)	176.3	203.1	2.65	<0.001
FCR (g/g)	5.24	5.21	0.24	0.522

^1^ polyacrylamide supplemented diet

^2^ Body weight

^3^ Voluntary feed intake

^4^ Daily body weight gain

^5^ Feed conversion ratio

### Impact of supplementation of polyacrylamide on nutrient digestibility of lambs

As shown in Table 3, PAM supplementation increased the apparent digestibility of DM (by 7.3%), OM (by 5.4%), CP (by 6.4%), cellulose (by 9.6%), energy (by 4.3%), calcium (by 8.5%), phosphorus (by 18.7%) (all above *P <* 0.01) and hemicellulose (by 22.6%) (*P <* 0.05) at the age of 95–103 days and that of DM (8.7%), OM (7.9%), CP (7.7%), cellulose (21.4%), energy (6.9%), calcium (9.0%), phosphorus (18.4%), and hemicellulose (11.6%) (all above *P <* 0.01) at the age of 210–218 days.

**Table 3:** The effect of supplementation with polyacrylamide on the apparent digestibility and the amounts of digested nutrients in the diet of lambs.

Days of age	95~103				210~218			
Treatments	Control	^1^PAM	SEM	*P value*	Control	PAM	SEM	*P value*
Digestibility (%)								
Dry matter	61.60	66.47	0.41	0.002	64.52	70.50	0.38	<0.001
Organic matter	65.63	69.16	0.40	0.001	67.29	72.63	0.40	0.005
Crude protein	65.94	70.15	0.37	0.001	66.74	71.91	0.52	<0.001
Cellulose	25.72	28.20	0.18	0.001	54.56	60.91	0.36	<0.001
Hemicellulose	29.38	36.00	0.33	<0.001	37.26	45.24	0.40	0.001
Energy	73.15	76.32	0.43	0.007	69.44	74.25	0.39	0.001
Calcium	59.61	64.65	0.38	0.001	65.87	71.83	0.36	0.001
Phosphorus	39.16	46.49	0.42	0.001	40.99	48.52	0.46	<0.001
Digested amounts (g or MJ/lamb/day)								
Dry matter	515.2	624.4	3.73	<0.001	855.8	1075.1	6.39	<0.001
Organic matter	502.4	594.6	2.89	<0.001	823.8	1022.3	6.55	<0.001
Crude protein	85.4	101.3	0.91	<0.001	110.0	135.9	1.08	<0.001
Cellulose	24.8	31.3	0.28	<0.001	125.9	162.9	1.17	<0.001
Hemicellulose	21.6	30.6	0.41	<0.001	66.4	93.4	0.98	<0.001
Energy	10.71	12.55	0.09	<0.001	15.01	18.46	0.09	<0.001
Calcium	4.65	5.63	0.03	<0.001	7.77	9.70	0.06	<0.001
Phosphorus	0.99	1.32	0.02	<0.001	1.61	2.19	0.02	<0.001

^1^ polyacrylamide supplemented diet

Additionally, as shown in Table 3, PAM supplementation increased the amounts of digested DM (by 20.4%), OM (by 18.4%), CP (by 18.6%), cellulose (by 26.2%), hemicellulose (by 41.7%), energy (by 17.2%), calcium (by 21.1%), and phosphorus (by 33.3%) (all above *P <* 0.01) at the age of 95–103 days and the DM (24.9%), OM (24.1%), CP (23.5%), cellulose (29.4%), hemicellulose (40.7%), energy (23.0%), calcium (24.8%), and phosphorus (36.0%) (all above *P <* 0.01) at the age of 210 to 218 days.

### Metabolism of nitrogen, calcium, and phosphorus

Results of metabolism of nitrogen and Ca and P are presented in Table 4. Results showed that PAM supplementation increased the nitrogen retention and nitrogen retention rate by 30.3% and 16.8% (*P <* 0.01) in lambs aged 95–103 days and by 38.6% and 20.9% (*P <* 0.01) in lambs aged 210–218 days, respectively. Lambs fed the diet supplemented with PAM produced fecal and urinary excretions of nitrogen similar to those of the control lambs (*P >* 0.05).

**Table 4:** The effect of supplementation with polyacrylamide on the metabolism of nitrogen, calcium, and phosphorous in lambs.

Days of age	95~103				210~218			
Treatments	Control	^1^PAM	SEM	*P value*	Control	PAM	SEM	*P value*
Nitrogen								
Intake (g/lamb/day)	20.71	23.11	0.14	<0.001	26.37	30.23	0.08	<0.001
Fecal excretion (g/lamb/day)	7.06	6.90	0.08	0.358	8.77	8.49	0.15	0.071
Urinary excretion (g/lamb/day)	5.12	5.08	0.10	0.859	6.18	5.92	0.09	0.070
Retention (g/lamb/day)	8.54	11.13	0.11	<0.001	11.42	15.82	0.12	<0.001
Retention rate (%)	41.23	48.16	0.40	<0.001	43.3	52.33	0.373	<0.001
Calcium								
Intake (g/lamb/day)	7.81	8.71	0.01	<0.001	11.8	13.51	0.04	<0.001
Fecal excretion (g/lamb/day)	3.15	3.08	0.03	0.194	4.03	3.80	0.05	0.059
Urinary excretion (g/lamb/day)	0.29	0.28	0.02	0.678	0.37	0.38	0.03	0.842
Retention (g/lamb/day)	4.36	5.35	0.03	<0.001	7.40	9.33	0.04	<0.001
Retention rate (%)	55.86	61.40	0.37	0.001	62.72	69.05	0.33	0.001
Phosphorus								
Intake (g/lamb/day)	2.53	2.84	0.03	<0.001	3.94	4.52	0.03	<0.001
Fecal excretion (g/lamb/day)	1.54	1.52	0.02	0.360	2.32	2.33	0.03	0.742
Urinary excretion (g/lamb/day)	0.09	0.11	0.01	0.061	0.13	0.08	0.01	0.106
Retention (g/lamb/day)	0.90	1.20	0.01	<0.001	1.48	2.11	0.01	<0.001
Retention rate (%)	35.56	42.47	0.44	<0.001	37.68	46.66	0.34	<0.001

^1^ polyacrylamide supplemented diet

Results also explored that PAM supplementation increased calcium retention and its retention rate in lambs by 22.7% and 9.9% (*P <* 0.01), respectively, at the age of 95–103 days, and by 26.1% and 10.1% (*P <* 0.01), respectively, at the age of 210–218 days. Polyacrylamide supplementation increased phosphorus retention and its retention rate by 30.3% and 19.4% (*P <* 0.01) at the age of 95–103 days and by 42.6% and by 23.8% (*P <* 0.01) at the age of 210–218 days, respectively. The increments in both Ca and P retention were mainly due to the increase in intake.

### Slaughter performance

The results of slaughter performance parameters are shown in Table 5. Results of slaughter performance parameters explored that PAM supplementation increased the carcass weight, meat weight and lean weight by 24.5%, 25.5% and 30.6% (*P <* 0.01), respectively.

**Table 5:** The effect of supplementation with polyacrylamide on the slaughter performance of lambs.

Treatments	Control	PAM	SEM	*P value*
Live weight (kg)	44.68	50.30	0.73	0.001
Carcass weight (kg)	19.66	24.48	0.33	< 0.001
Dressing rate (%)	44.00	48.68	0.37	< 0.001
Meat weight (kg)	15.74	19.76	0.29	< 0.001
Meat weight rate (%)	29.58	34.31	0.34	< 0.001
Lean weight (kg)	13.22	17.26	0.33	< 0.001
Lean weight rate (%)	67.22	70.48	0.53	0.020
Fat weight (kg)	2.52	2.50	0.11	0.887
Bone weight (kg)	3.92	4.72	0.06	< 0.001
Meat to bone ratio (%)	4.02	4.19	0.06	0.065
Loin muscle area (cm^2^)	16.52	18.58	0.19	< 0.001

As shown in Table 6, PAM supplementation increased the fresh weights of liver, leg muscle, *Longissimus dorsi* muscle, and rumen tissues by 18.6%, 26.5%, 33.9%, and 14.3% (*P <* 0.01), respectively, but not the weight of kidneys compared with those of the control. Supplementation of PAM in the diet decreased the CP contents of *Longissimus dorsi* by 8.4% (*P <* 0.05) but increased the DNA content of leg muscle by 20.0% (*P <* 0.05). It was also observed that supplementation of PAM in the diet increased the contents of DM and OM in fresh liver tissue but decreased the contents of DM, OM, CP, and DNA in fresh kidney tissue (*P<0*.*05*). Furthermore, the RNA contents were significantly increased in all fresh tissues except the liver tissues.

**Table 6:** Effects of PAM on the fresh weight and the contents of DM, OM, CP, DNA, and RNA in the internal organs and muscles of lambs.

Treatments	Control	PAM	SEM	*P value*
Weight of fresh tissue (g)				
Liver	523.40	620.80	6.50	< 0.001
Kidneys	98.60	100.20	3.26	0.412
Leg muscle	6512.40	8238.00	123.81	< 0.001
*Longissimus dorsi*	1336.60	1790.00	37.13	< 0.001
Rumen	544.00	621.80	15.50	< 0.001
Dry matter (mg/g fresh tissue)				
Liver	266.94	282.32	3.10	0.006
Kidneys	269.96	224.19	8.00	< 0.001
Leg muscle	218.96	225.48	6.24	0.234
*Longissimus dorsi*	235.36	243.16	9.98	0.370
Rumen	145.14	146.10	10.36	0.895
Organic matter (mg/g fresh tissue)				
Liver	253.03	276.79	8.35	0.231
Kidneys	243.42	221.17	7.89	< 0.001
Leg muscle	213.70	217.39	5.64	0.314
*Longissimus dorsi*	228.04	239.66	4.65	0.019
Rumen	126.01	138.44	4.37	0.009
Crude protein (mg/g fresh tissue)				
Liver	158.52	155.45	1.77	0.140
Kidneys	173.64	148.04	5.21	0.001
Leg muscle	160.93	168.89	3.00	0.077
*Longissimus dorsi*	183.83	168.34	3.85	0.004
Rumen	163.23	170.74	4.44	0.001
DNA (μg/g fresh tissue)				
Liver	363.79	357.00	4.24	0.183
Kidneys	493.97	360.22	17.59	< 0.001
Leg muscle	78.05	93.69	4.50	0.001
*Longissimus dorsi*	99.68	96.01	4.98	0.062
Rumen	170.47	153.71	5.01	0.001
RNA (μg/g fresh tissue)				
Liver	1434.06	1450.18	22.66	0.643
Kidneys	1555.62	1300.30	40.93	< 0.001
Leg muscle	2127.25	2290.05	42.00	0.021
*Longissimus dorsi*	1592.83	1762.89	39.21	0.004
Rumen	1274.15	1147.14	34.92	< 0.001

## Discussion

In recent decades, surfactants have been used in bioreactors to facilitate the enzymatic hydrolysis of lignocellulose to produce bioethanol and biobutanol [25, 26]. Generally, nonionic surfactants, such as Tween and polyethylene glycol, has been reported to reduce the interaction between enzymes and lignin and beneficial effect on fiber degradation. It has also been reported that cationic surfactants inhibit cellulase activity and thus negatively affect the enzymatic hydrolysis of cellulose [27]. Although the role of anionic surfactants in cellulose degradation has not been consistently concluded, however, studies have found that some anionic surfactants, such as sodium dodecyl sulfate (SDS), could promote cellulose conversion and delignification [28, 29]. The biochemical process of producing bioenergy from lignocellulose has many similarities with rumen fermentation [30]. Studies have found that an appropriate amount of anionic surfactant can reduce the number of rumen ciliates, lower ruminal pH and ammonia nitrogen concentration, increase *in vitro* gas production and microbial protein content, and therefore have beneficial effects on rumen fermentation [31, 32]. In addition, anionic surfactant supplementation can also work synergistically with *Lactobacillus plantarum*, *Pediococcus acidilactici*, and *Enterococcus faecium* to improve the fermentation characteristics and the degradation rate of NDF in barley silage [33]. Based on results of invitro studies, it could be speculated that the supplementation of the anionic surfactant in the diet of ruminants may have positive impact on nutrient intake, digestibility, growth and carcass parameters.

Dietary PAM inclusion in the diet of lamb increased VFI, an effect similar to that reported previously [9, 14] for other anionic surfactants supplementation in animal’s diet. For example, dietary supplementation with DOC at a dose of 0.8 g/kg diet has been reported to increase VFI by 23.6% [14] and 30.7% [9] in sheep. Previous studies has observed a dose dependent effect on VFI by supplementation of PAM in the diet of animals and reported that PAM supplementation at doses of 1.0, 2.0, 3.0 and 6.0 g/kg diet increased VFI by 8.8%, 17.9%, 8.2% and 5.5% [19], respectively. Based on the results of does dependent response a dose of 2.0 g/kg diet of PAM was selected in the current experiment and surprisingly an improved VFI was observed in the current study.

The improved digestibility results are similar with the findings of the previous studies who reported that PAM supplementation in the diet of the dairy cows improved the nutrient digestibility [34]. In a previous study, surfactant inclusion was found to increase in vitro DM and OM digestibility of low-quality roughages [35]. Similarly, another invitro study has explored the treatment of barely silage with anionic reagent sodium dodecyl sulfate improve NDF degradability in the silage [33]. Our findings are in agreement with these previous results and the improvement of total tract nutrient digestibility might be related to the increase of the ruminal effective degradability of nutrients. Previous in vivo study has explored increases in fiber-degrading-related bacteria and activities of enzymes in the rumen by supplementation of surfactant in the diet of ruminants [19] and in the current study higher digestibility could be attributed to increases in fiber-degrading-related bacteria and activities of enzymes. The other possible reason of higher digestibility could be explained by better fermentation and antiprotozoal effects of surfactants [12, 36]. It has been reported previously that surfactant detergents have antiprotozoal effects on the rumen of sheep [36] and a reduction of 33.7% of protozoal count in sheep has been reported by supplementation of PAM in the diet of sheep [19]. Supplementation of PAM in the sheep diet not only reduce the protozoal count but also increase the total bacterial number and activities of fiber-degrading enzymes [19] that could also be one of the reason of higher digestibility in the current study. Previous studies have clearly mentioned that higher fiber-degrading enzymes activities in the partially fauna-depleted rumen than those in both normal and fauna-free rumens [12, 37] and higher nutrient digestibility of partially fauna-depleted rumens [14, 38]. Based on results of the current study and results of the previous studies it could be speculated that PAM supplementation could be good strategy to reduce partial reduction in the protozoal count [39], increase fiber degrading bacteria especially *R*. *flavefaciens*, *F*. *succinogenes* and *B*. *fibrisolvens* [19], increase in nutrient digestibility in ruminants.

Higher body weight gain in lambs with the supplementation of PAM could easily be explained by the higher VFI and nutrient digestibility in lambs. In the current study, the more profound effect of increasing VFI in the older lambs suggested that PAM supplementation have more positive effect in fully developed rumen as compared to predeveloped rumen. Moreover, higher DBG, nitrogen retention and digestibility of OM, cellulose and energy at the age 210 to 218 days lambs as compared to 95 to 103 days old lamb could also explained by theory of fully developed rumen as described in higher VFI during these days of life. Higher weight gain and better carcass characteristics by supplementation of PAM in the diet of lambs are also in agreement with previously published report who reported that supplementation with the polymer gel PH20 increased body fat deposition and persistence of milk yield in dairy cows [40], possibly because PH20 has similar physical-chemical properties to PAM, therefore, in the current study better weight gain and better carcass yield by supplementation of PAM in the diet of lamb could easily justified. To our knowledge, no study has been conducted to evaluate the effects of PAM supplementation on carcass characterristics, therefore, it is suggested to conduct further studies to explore the possible mechanisms of better carcass characteristics by supplementation of PAM in the diet of lambs.

It is worth noting that PAM supplementation did not change the feed conversion ratio in lambs in the present study, however PAM supplementation has changed the BWG that shows that both higher VFI and digestibility are the reason of higher BWG in PAM supplemented lambs.

PAM is generally considered safe, but acrylamide (AA), as a free monomer in PAM products, is considered toxic [41]. According to the EU, the threshold of AA in food should be between 50 and 850 μg/kg for example 50 μg/kg for wheat-based bread, 350 μg/kg for biscuits and wafers, 500 μg/kg for French fries (ready-to-eat), 750 μg/kg for fried potato chips, and 800 μg/kg for ginger bread has been recommended [42]. AA in food grade PAM is below 250 μg/kg. Therefore, in this experiment, the AA level in the diet for sheep with 50 kg BW was below 500 μg/kg, which was similar to that of french fries (ready-to-eat). European Food Safety Authority has declared the BMDL_10_ (benchmark dose level 10) for AA in human as 170 μg/day/kg BW [43, 44]. In this experiment, the amount of AA taken by sheep (with BW of 50 kg, 1.5 kg diet per day) through feed was below 15 μg/day/kg BW, therefore, it could be speculated that PAM level was safe for lamb and lambs meat could be safe for human consumption.

## Conclusion

In conclusion, the present study shows that dietary PAM supplementation improves the voluntary feed intake, total digestive tract digestibility of nutrients, growth performance and carcass characteristics of lambs. These results further imply that PAM could be used as a potential feed additive in ruminant.

## Supporting information

S1 File (RAR)
